# Supplementary material for: Chromothripsis during telomere crisis is independent of NHEJ, and consistent with a replicative origin
Source: Genome Res. 2019 May;29(5):737–49. doi: 10.1101/gr.240705.118 (PMC6499312; doi:10.1101/gr.240705.118)
Supplement: Supplemental Material [file supp_gr.240705.118_Supplemental_file_1.zip › contigs/annotated_contigs/DB54/contig.2.DB54_length_699_mean_cov_7.29613733906.docx]

**DB54_length_699_mean_cov_7.29613733906**

```
GTTCTCAGCCCTGCTGGCAGCCCTGGGCTATGAGCTGCAGGGGTACTGTCTGTCCTGGGCCCTAAATCTGTCCCAACTGCCTTGTGAGA
 >chr19:17974329-17974708 + E=3e-213
CACAGGCCTCCCAGGCTCCCCTGCTGAGTCTGGACCAGGGAGAATCTCCCTAGATAGTGGGGTTGGCGTTCACTCCCTGGGGAGTCTCC

TAAGTCTCTGGTTCCCAAAGTTTTCAGAGTAGACACAAGTGGGCACCCGAGGGGCGCTCTCTGCTGCTTGGGGGCTGTTTCCCTTTCAC

TGGTTGGGGGCCTCTTAGATGACGCCCTCATCTTGCCCAAGGAGGTCAAGGACTCACCACAGGTACTCATCTCTGGGAAACTGCCTCAG

GCCTGGAAAATTCCAGGCATG|AC|GCTGGCCTGTGTGTGCGCCCTGCTGGTGTGGCACCCCGCCGGCCCCGGCGAGAAGGTGGTGCGC
                      >chr19:17837414-17837736 + E=6e-176
GTGCTGTTCCCCGGTTGCACCCCGCCCGCCTGCCTCCTGGACGGCCTGGTCCGCCTGCAGCACTTGAGGTTCCTGCGAGAGCCCGTGGT

GACGCCCCAGGACCTGGAGGGGCCGGGGCGAGCCGAGAGCAAAGAGAGCGTGGGCTCCCGGGACAGCGCGAAGAGAGAGGGCCTCCTGG

CCACCCACCCTAGACCTGGCCAGGAGCGCCCTGGGGTGGCCCGCAAGGAGCCAGCACGGGCTGAGGCCCCACGCAAGA
```
